# Characterization of moderate tendinopathy in ex vivo stress-deprived rat tail tendons

**DOI:** 10.1186/s12938-019-0673-y

**Published:** 2019-05-08

**Authors:** Leila Jafari, Martin Savard, Fernand Gobeil, Eve Langelier

**Affiliations:** 1 Department of Mechanical Engineering, Université de Sherbrooke, Sherbrooke, QC J1K 2R1 Canada; 2 Department of Pharmacology-Physiology, Université de Sherbrooke - Campus de la santé, Sherbrooke, QC J1H 5N4 Canada

**Keywords:** Stress deprivation, Tendinopathy, Biomechanical properties, Immunohistochemistry properties, Pathohistology, Degradation, Rat

## Abstract

**Background:**

Stress deprivation (SD) has frequently been used as a model to study tendinopathy. Most of these studies have investigated either short-term (early tendinopathy) or long-term SD (advanced tendinopathy), while the transient mid-term SD has been given less attention. Therefore, the main objective of this study was to characterize mid-term SD.

**Methods:**

To this end, live, healthy rat tail tendons (RTTs) were harvested and cultured without mechanical stress and then were divided into five groups based on their culture time (fresh, 2-day SD, 4-day SD, 6-day SD, and 10-day SD). For each group, the tendons were subjected to traction testing and pathohistology, immunohistochemistry, and viability assays.

**Results:**

Our results showed that 4 days of SD resulted in moderate pathological changes in RTTs. These changes included increases in the space area between fibers, cell density, and fiber tortuosity as well as a decrease in collagen density and elongation of cell nuclei. No changes in the stress at failure of tendons were observed at this time point.

**Conclusions:**

This simple ex vivo model is expected to be useful for studying the progression of tendinopathy as well as for testing potential mechanobiological or pharmacological therapy strategies to stop or reverse the progression of the pathology.

## Background

Tendinopathy is a frequent health problem that accounts for over 65% of work-related musculoskeletal disorders [1]. It is important not only to study and understand tendinopathy because of its prevalence, but also to find an optimal treatment for the disease. Many treatment modalities have been proposed for tendinopathy (e.g., nonsteroidal anti-inflammatory drugs, stem cell or gene therapy, eccentric exercises, laser therapy). However, there is very little support of the efficacy of these treatments [2]. In vitro, ex vivo, and in vivo models of tendinopathy are part of a multiscale approach to understand and develop treatments for tendon disorders [3, 4]. Stress deprivation (SD) of tendon tissues has been frequently used to develop such models [5–7]. SD can be categorized as short term or long term based on the investigation time points and the obtained results. For example, studies using short-term ex vivo SD models at 24 h, 48 h, and 72 h [5–7] observed increases in apoptosis, cell roundness, and MMP-13 gene/protein expression as well as a decrease in fiber density. In addition to these early changes, long-term studies at 6 days, 12 days [8], 1 week, 2 weeks, and 8 weeks [9–12] observed increases in collagen fiber waviness and disorientation, a decrease in biomechanical properties, and an increase followed by a decrease in cell density. Table 1 summarizes the results from previous ex vivo studies on stress-deprived tendons. These studies allowed characterization of short-term (early) and long-term (advanced) tendinopathy models. However, there is still a need to study different time points within this interval to better understand the transition of the tissue condition during the progression from early to advanced tendinopathy. This transition state shall be referred to as moderate tendinopathy.

**Table 1:** Data extracted from ex vivo SD studies

References	Model	Culture duration	Results
Short-term SD (early tendinopathy)			
Arnoczky et al. [5]	Rat, adult, tail tendon	1, 2, 3 days	*Biomechanical* No change in maximum stress *Histological* Cell roundness *Immunohistochemistry* Increase in MMP-13 expression
Egerbacher et al. [6]	Rat, adult, tail tendon	24 h	*Biomechanical* N/A *Histological* Decrease in collagen fiber density *Immunohistochemistry* Increase in MMP-13 expression and MMP-13 protein synthesis
Egerbacher et al. [42]	Rat, adult, tail tendon	24 h	*Biomechanical* N/A *Histological* Increase in cell apoptosis *Immunohistochemistry* N/A
Gardner et al. [7]	Rat, 6 months old, tail tendon	24, 48, 72 h	*Biomechanical* N/A *Histological* N/A *Immunohistochemistry* Increase in MMP-13 expression
Long-term SD (advanced tendinopathy)			
Abreu et al. [9]	Rat, male, adult, tail tendon	1 week	*Biomechanical* Decrease in elastic modulus *Histological* N/A *Immunohistochemistry* Decrease in GAG^a^ content
Arnoczky et al. [10]	Rat, adult, tail tendon	7 days	*Biomechanical* Decrease in ultimate stress, tensile modulus and strain at ultimate stress *Histological* Less dense collagen fiber packing *Immunohistochemistry* Increase in MMP-13 expression and MMP-13 protein synthesis
Hannafin et al. [11]	Canine, adult, mixed age and sex, flexor digitorum tendon	8 weeks	*Biomechanical* Decrease in tensile modulus *Histological* Decrease in cellularity Cell roundness Mild increase in collagen crimps *Immunohistochemistry* N/A
Lavagnino et al. [12]	Rat, 13 months old, tail tendon	21 days	*Biomechanical* Decrease in ultimate tensile strength and tensile modulus *Histological* No change in collagen fiber density *Immunohistochemistry* N/A
Wang et al. [8]	Rabbit, female, 15 weeks old, Achilles tendon	6,12 days	*Biomechanical* N/A *Histological* Increase in cell apoptosis *Day 6*: increase in cell density; *Day 12*: decrease in cell density Cell roundness Loose collagen fibers Increase in space between collagen fibers Wavy and disoriented fibers *Immunohistochemistry* N/A

N/A stands for data not available

^a^Glycosaminoglycans: an ECM constituent

In addition to studying early and advanced tendinopathy, studying moderate tendinopathy is crucial to understand all stages of damage that the tendons experience during lesion development. Moderate-stage tendinopathy becomes even more important when investigating the potential efficacy of treatments, because early-stage human tendinopathy is often asymptomatic [3, 13] and patients often do not seek medical attention. Advanced tendinopathy, however, usually requires invasive surgery for treatment [14]. Therefore, moderate tendinopathy would be a better time for studies that are designed to estimate the effectiveness of new pharmacotherapies.

Therefore, in the present study, we designed a moderate SD model consisting of a 10-day SD experiment with measurements taken every 2 days until day 6, and then at day 10 for the histological, immunohistochemistry, and biomechanical end-point assays. We hypothesized that moderate tendinopathy: (1) occurs at a time point between 3 and 6 days SD; and (2) is characterized by a progressive increase in cell apoptosis, cell roundness, and MMP-13 level, a progressive decrease in fiber density, and mechanical properties as well as a reversal of cell density trend, i.e., a decrease following an initial increase.

## Methods

### Animals

Experiments were carried out on male Sprague–Dawley rats weighing 500–800 g (4 to 6 months old) (Charles River; St. Constant, Québec, Canada). Animal housing and the experimental protocol (protocol #EL2013-01) were carried out in accordance with regulations set by the Canadian Council of Animal Care Committee and were approved by the Animal Care Committee of the University of Sherbrooke (CIPA/CFPA-FMSS).

### RTT extraction

**Fig. 1:**
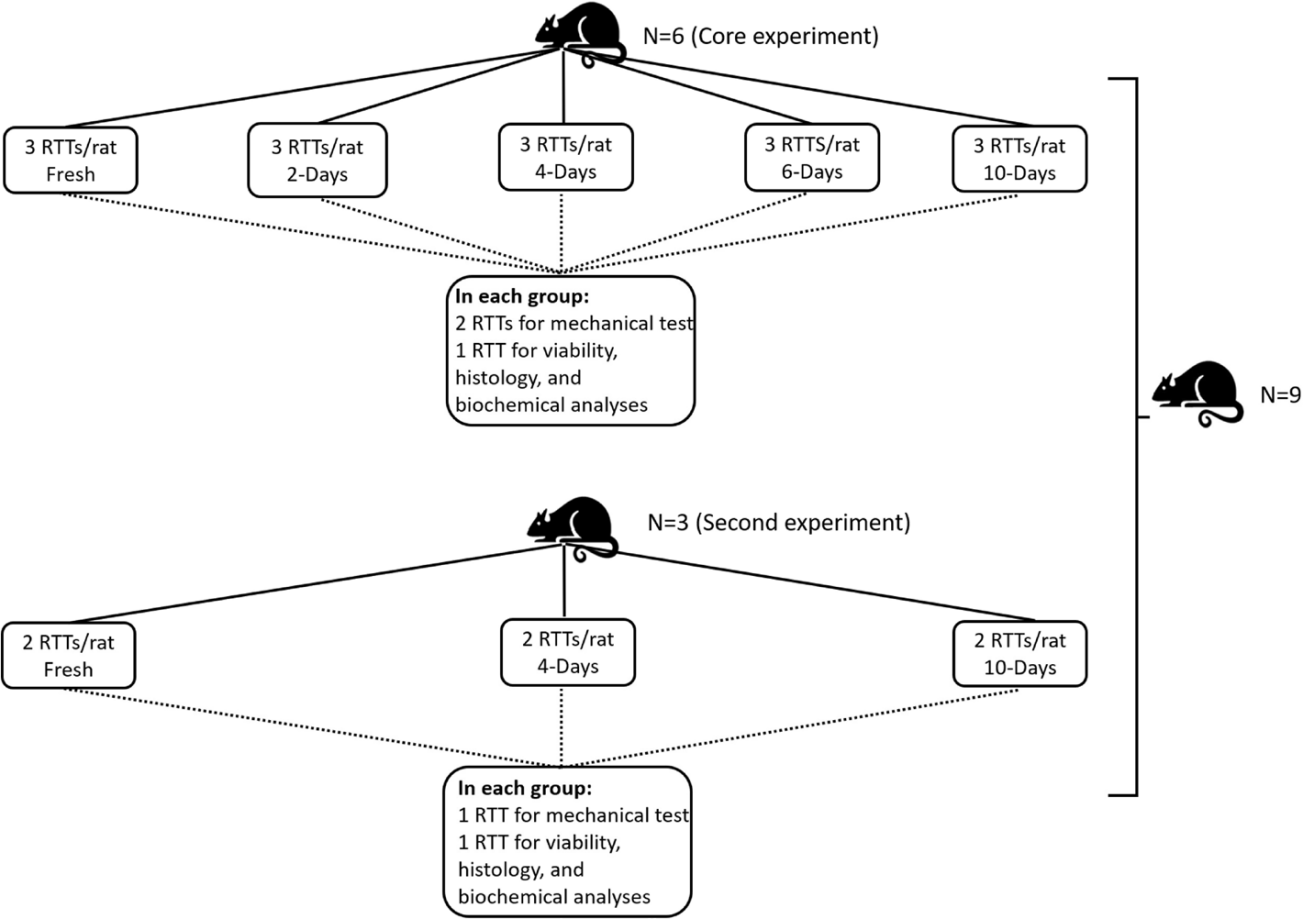
Number and distributions of tendons from each rat. RTT stands for rat tail tendon, and 2 days, 4 days, 6 days, and 10 days stand for 2, 4, 6, and 10 days of stress deprivation, respectively. In the core experiment, there were three tendons in each group, two for biomechanical analysis, and one for viability, histology, and immunohistochemistry analyses. In the secondary experiment, there were two tendons per group: one for biomechanical analysis and the other for viability, histology, and immunohistochemistry analyses

Tendon isolation and preparation were conducted as described in a previous study [15]. The distribution of rats and tendons for biomechanical testing and viability, histology and immunohistochemistry analyses are shown in Fig. 1. All manipulations were performed in a cold Dulbecco’s phosphate-buffered saline (D-PBS) solution (311-410-CL; Wisent Inc., St-Bruno, Canada) containing 1 g/L glucose (609-037-EL; Wisent Inc.) and 1% antibiotic–antimycotic (15240-062; Invitrogen, Burlington, Canada). Following isolation, tendon cross-sectional areas were evaluated using an optic micrometer and a stereomicroscope [16]. Briefly, the tendons were maintained in the saline solution to avoid dehydration and rotated along their longitudinal axis. Images of specimen projections were captured at 10° angular increments and the tendon edges were localized within a local reference frame using a contrast-based image analysis algorithm. The cross-sectional areas were estimated using a profile reconstruction algorithm. The tendons were then washed five times under sterile conditions. Afterward, they were transferred to tissue culture flasks containing DMEM solution (12800-017; Invitrogen, Burlington, Canada) supplemented with 3.7 g/L of sodium bicarbonate (600-105-CG; Wisent Inc.), 10% FBS (090150; Wisent Inc.), and 1% antibiotic–antimycotic.

### RTT incubation

Tendons were divided into five groups in the core experiments and three groups in the secondary experiments based on culture time (Fig. 1). In each experiment, fresh tendons were used as controls to the SD tendons. SD tendons were incubated under tissue culture conditions (37 °C, 95% humidity, 5% CO_2_, sterile environment) for 2, 4, 6, or 10 days. In the core experiments, there were three tendons in each group, two for biomechanical testing and one for the viability, pathohistological and immunohistochemistry studies. In these experiments, the biomechanical results from the two tendons were averaged for each group. In the secondary experiment, there were two tendons per group, one for biomechanical characterization and one for the viability, pathohistological and immunohistochemistry studies. The media were changed every 2 days.

### RTT biomechanical characterization

Biomechanical tests were conducted on live tissues at the end of their culture time (i.e., 0 (control), 2, 4, 6, or 10 days). At the time of mechanical testing, the ends of the tendon were wound around cylinder-shaped anchors and allowed to dry briefly on the top face of the anchors. A small drop of ethyl cyanoacrylate (10300; Krazy Glue, Columbus, OH) was applied to the portion of the tendon at the top of the anchor. The tendons were then transferred to a custom-manufactured bioreactor [17]. The specimens were subjected to the following testing protocol: The initial zero-strain reference was defined by achieving a tension load of 3 g at equilibrium. Preconditioning was performed with a series of 120 sinusoidal waves at two different amplitudes (60 cycles at 1% strain; 60 cycles at 2% strain) at 1 Hz. The final zero-strain reference was defined by again reaching a tension load of 3 g at equilibrium. Afterward, the tendons were subjected to a traction test (at a strain rate of 0.5%/s). Maximum engineering stress before failure was used as a measure of tendon strength. Data are reported as normalized to day 0.

### Viability test

Viability tests were conducted on fresh and stress-deprived RTT at the end of their culture time (i.e., 0, 2, 4, 6, or 10 days). Viability of cells from the fresh and cultured tendons was assessed using the LIVE/DEAD Viability/Cytotoxicity Kit for mammalian cells (L3224; Invitrogen, Carlsbad, CA) according to the manufacturer’s instructions. Tendons were washed in D-PBS to remove any serum and then incubated with ethidium homodimer-1 (1 µM for 45 min), followed by treatment with calcein AM (1 µM for 40 min) in D-PBS at room temperature. The tendons were washed briefly in D-PBS prior to being mounted on glass slides. Green-fluorescing live cells and red-fluorescing dead cells were visualized within 45 min with an EVOS FL Auto microscope (Thermo Fisher Scientific, Massachusetts, USA). Images were captured with a digital camera. Control RTT samples, cultured for the same time and using the same media, were subjected to low static loading to avoid stress deprivation. One-and-a-half MPa was used as low static load. This loading amplitude was chosen based on the reported anticatabolic effect of low SL on tenocytes in the literature [18, 19] and our results from preliminary tests. This load was applied on tendons by attaching a stainless steel weight (adapted to each tendon cross-sectional area) to an approximately 11 cm long RTT. Control RTT samples showed the same level of cell viability as fresh tendon (data not shown).

### Histological characterization

Histological studies were conducted on RT tissues fixed in 10% neutral buffered formalin and embedded in either paraffin for hematoxylin and eosin staining or in OCT medium.

Paraffin-embedded tissues were longitudinally sectioned into 6 µm-thick sections and were processed in hematoxylin and eosin (H&E) stain for light microscopy. Images of each sample were captured using whole slide imaging with a Nanozoomer (Hamamatsu Photonics K.K., Hamamatsu city, Japan) in visible mode. The 20× images were analyzed using the following five parameters: fiber density, space area between fibers, fiber tortuosity, cell number, and elongation of nucleus.

The two first parameters of the extracellular matrix were evaluated as described previously [20]. For each micrograph, three regions of interest (ROIs) were captured taking care not to include damages caused by the cutting blade. The ROIs were then analyzed with Image-Pro Plus software (version 6.0). Data are presented as normalized to day 0.

### Space area between fibers

For each ROI, the areas of the spaces between fibers were also evaluated in terms of pixel numbers and then averaged. Data are reported as normalized to day 0.

### Fiber density

The background density was found by contrast and separated into two categories: background (red) and fibers (black). The fiber density was calculated as:

1 $${\text{Fiber density }}\left( \% \right) = \left( {1 - \frac{\text{number of pixels of background between fibers}}{\text{number of pixels of ROI}}} \right) \times 100\% .$$

Results from the three ROIs were averaged. Data are presented as normalized to day 0.

### Fiber tortuosity

For collagen fiber tortuosity analysis, digital images of H&E stained slides were randomly numbered for blind analysis. Fiber tortuosity was scored between 0 and 3 using a semiquantitative scale similar to the Bonar scoring method [21]. 0 was assigned to images that showed the least collagen tortuosity (collagen fibers were mostly aligned in the longitudinal direction of tendon), whereas 3 was assigned to images with the waviest collagen fibers.

### Cell number

Cell number was evaluated as:

2 $${\text{Cell number}} = \frac{\text{number of cells}}{\text{tendon longitudinal area}}.$$

Using a grid with a known scale bar, the number of cells was counted, and the tendon longitudinal area was calculated. Only cells that had crossed the right or top sides of the grid, either completely or partially, were counted. Data are reported as normalized to day 0.

### Elongation of nucleus

Longitudinal cryosections of formalin-fixed tissues of 20 µm thickness were stained with DAPI (DAPI, Sigma Aldrich, 62248, 1 µg/mL). Images of blue nuclei were captured using whole slide imaging with the Nanozoomer. The 40× images were analyzed using Image-Pro Plus software. The nucleus shape was recognized by contrast, and its elongation was evaluated as:

3 $${\text{Elongation of nucleus }} = \frac{{{\text{perimeter of nucleus}}^{2} }}{{4 \times \pi \times {\text{Area of nucleus}}}}.$$

A value of 1 indicates a perfect circle, whereas higher values indicate more elongated shapes. Data are reported as normalized to day 0.

### Immunofluorescence staining of MMP-13 protein

MMP-13 was examined because it is equivalent to MMP-1 (interstitial collagenase) in human and because its expression has been reported to be increased in pathological human tendons [10]. Immunohistochemical studies of MMP-13 were conducted on tissue samples that had been previously fixed in 10% neutral buffered formalin (032-060, Fisher Scientific, Company LLC, Kalamazoo, MI) and embedded in the optimal cutting temperature (OCT)-embedding medium. Embedded tissues were longitudinally cut into 20 μm sections using a cryostat (Thermo Scientific, Cheshire, UK). Sections were incubated in 0.1 M glycine (56-40-6, Roche diagnostic GmbH, Mannheim, Germany)/D-PBS for 10 min to wash away the mounting medium used in the tissue sectioning. Sections were incubated in D-PBS/0.1% Triton (T8787, Sigma Aldrich, Merck KGaA, Darmstadt, Germany)/2% bovine serum albumin (BSA) (A7030, Sigma Aldrich, Merck KGaA, Darmstadt, Germany) at room temperature for 30 min inside a humid chamber. They were then washed once in 0.1 M glycine/D-PBS and five times in D-PBS. After washing, sections were incubated with the rabbit polyclonal anti-MMP-13 antibody (ab39012, Abcam, Cambridge, UK, 20 µg/µL; all antibodies are diluted in D-PBS/0.05% Triton/1% BSA) and DAPI (62248, Sigma Aldrich, Oakville, Canada, 1 µg/mL) at room temperature for 1 h. Sections were washed five times in D-PBS and were incubated with the secondary antibody (A11034, Alexa Fluor 488 goat anti-rabbit IgG, Life Technologies, Carlsbad, California, USA; 20 µg/µL) at room temperature for 1 h. They were washed in D-PBS, mounted on microscopy slides with mounting media (F4680, Sigma Aldrich, Oakville, Ontario, Canada) and sealed with nail polish. Negative controls included sections incubated with the rabbit isotype IgG antibody, (X0903, Agilent Dako, Santa Clara, US; 20 µg/µL, diluted in D-PBS/0.05% Triton/1% BSA) without being exposed to the primary antibody. Green (MMP-13 proteins) and blue (nucleus) fluorescence images were captured with an EVOS FL Auto microscope (Thermo Fisher Scientific, Massachusetts, USA).

The 20× images of MMP-13 protein were analyzed using Image-Pro Plus software to quantify the intensity of MMP-13. Three ROIs focusing on the central zones of RTT were captured from each image. The MMP-13 intensity was calculated as the sum of the density of signals in each pixel from each ROI. The results from the three ROIs were averaged. Data are reported as normalized to day 0.

### RT-PCR analysis of MMP-13 mRNA transcript expression

In preliminary experiments, total RNA extraction from frozen RTT (*n* = 5 samples/condition) was performed using the TRIspin method [22], which combines the Trizol method with the RNeasy Total RNA Kit (Qiagen). RNA integrity was assessed with an Agilent RNA 6000 Nano LabChip according to the manufacturer’s protocol. Two micrograms of RNA from each sample was converted to cDNA using the Superscript First-strand RT-PCR Kit (Invitrogen) with random primers that were then subjected to PCR amplification using the Platinun Pfx Polymerase Kit (Invitrogen). To ensure the absence of genomic DNA contamination, RT reactions were also conducted in the absence of reverse transcriptase (-RT). Comparative quantifications of MMP-13 expression levels at each time point were performed by quantifying their respective PCR products using an Agilent DNA 1000 LabChip and expressed as ratios of the amount of each MMP amplicon over that of the corresponding 18S [23].

### Statistical analyses

Data are represented as the mean ± standard error of mean (SEM). A non-normal distribution was assumed for the data, since the sample size in each group was too small for a reliable normality test. Biomechanical, pathohistological, and immunohistochemistry results were compared using nonparametric Wilcoxon matched-pairs signed rank tests (GraphPad prism, 7.03) or one-median Wilcoxon tests (IBM SPSS, version 25.0), as appropriate. For doing multiple comparisons, first an exploratory test was performed to see if there was at least one difference between all the groups (dependent groups: Friedman test). After finding that this test was significant, a multiples comparisons test was conducted to see where the differences stands. Finally, a paired-Wilcoxon test was applied on each two groups at a time. Significance was set at *p* values < 0.050.

## Results

### Cross-sectional area

The cross-sectional areas of tendons were calculated to 0.036 ± 0.002 mm^2^.

### Cell viability

**Fig. 2:**
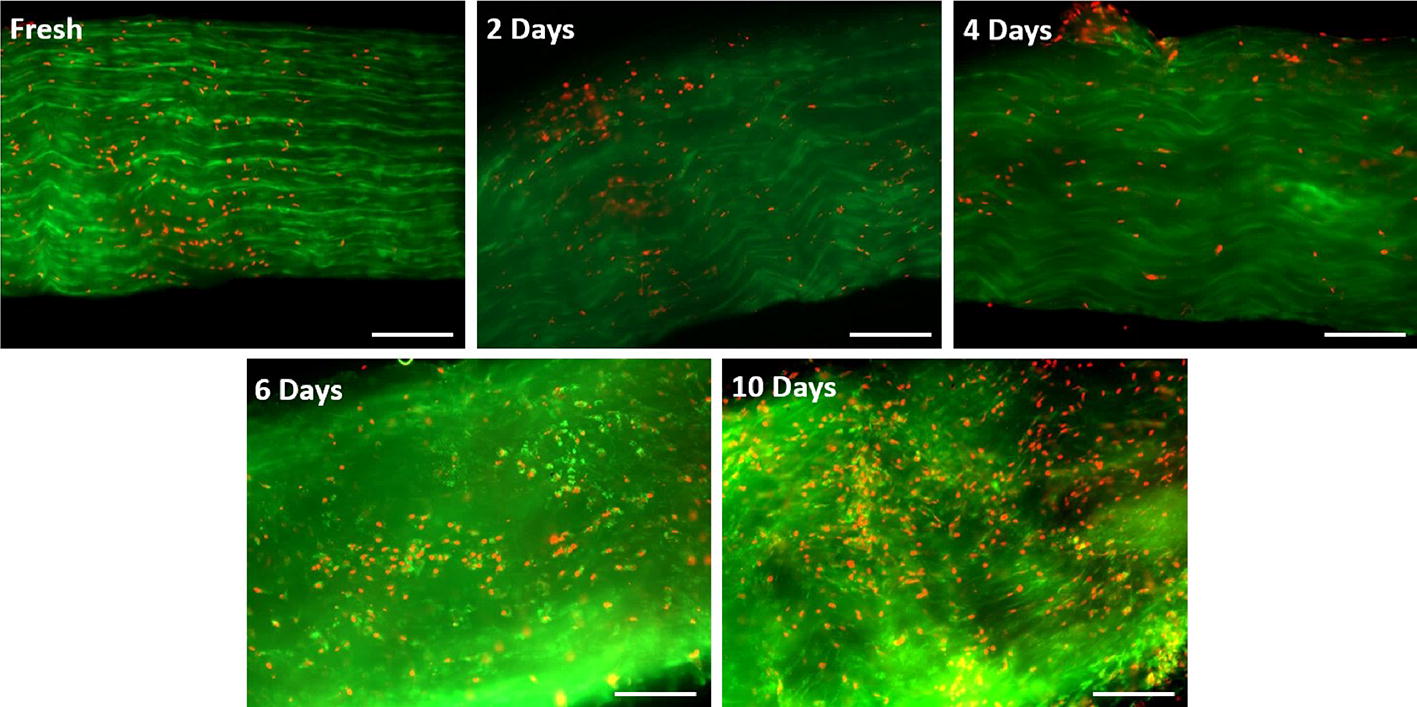
Representative fluorescent photomicrographs of tendons in the various groups (freshly isolated as well as 2-,4-, 6- and 10 days SD). Cell viability was determined using the live/dead viability/cytotoxicity kit. Calcein stains live cells green, while ethidium homodimer-1 stains dead cells red. Scale bar = 200 μm

Figure 2 presents viability images with surface cells in the focus area and deeper cells in the blur area. Under fluorescence microscopy, control (day 0) samples showed a high number of green cells and a few red cells, indicating a high level of viability (Fig. 2a). As the SD time increased, samples revealed a gradual increase in the number of red cells (Fig. 2b–e). Qualitative observations suggest that there was an increase in the rate of cell death after 6 and 10 days of SD compared to that at earlier time points (Fig. 2d, e).

### Biomechanical results

**Fig. 3:**
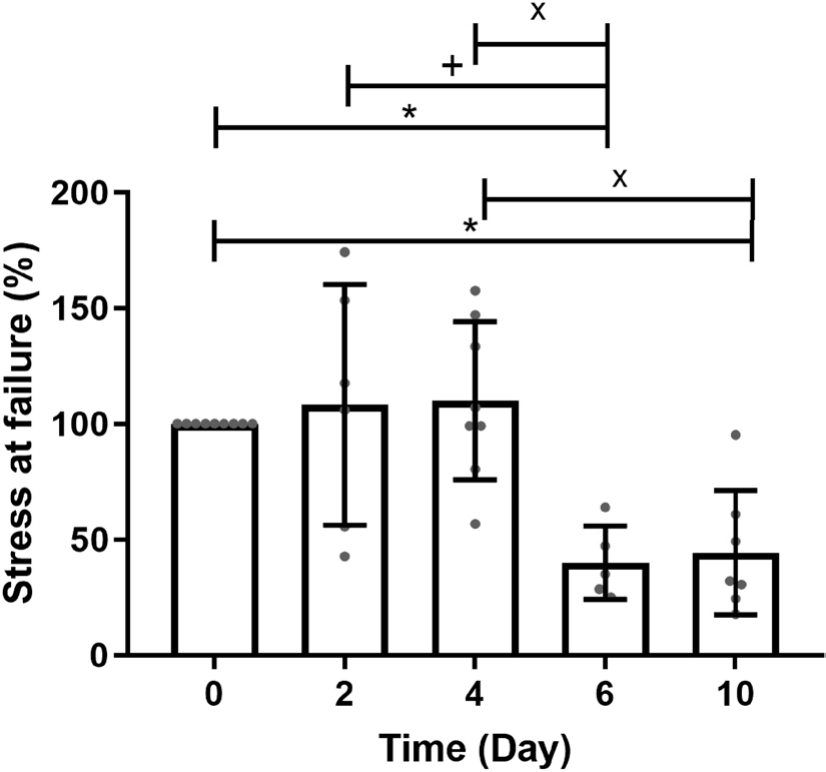
Changes in maximum stress at failure. Biomechanical testing was determined using custom-designed bioreactors applying axial strain. *p < 0.050 versus control fresh tendons; ^+^*p*<0.050 versus 2 days of stress deprivation; and ^×^*p* < 0.050 versus 4 days of stress deprivation. Nonparametric Wilcoxon matched-pairs signed rank tests were used to compare groups (except day 0) to each other. One-sample Wilcoxon tests were used to compare the groups to day 0

The maximum stress at failure of fresh tendons was evaluated to be 5.3 ± 1.0 MPa. The effect of SD on the maximum stress at failure of the RTTs is demonstrated in Fig. 3. There were no changes in the maximum stress at failure after 2 and 4 days of SD compared to that for the day 0 control. However, at 6 and 10 days of SD, there was a statistically significant decrease in the maximum stress at failure to approximately 40% of the initial stress at day 0.

### Histology

**Fig. 4:**
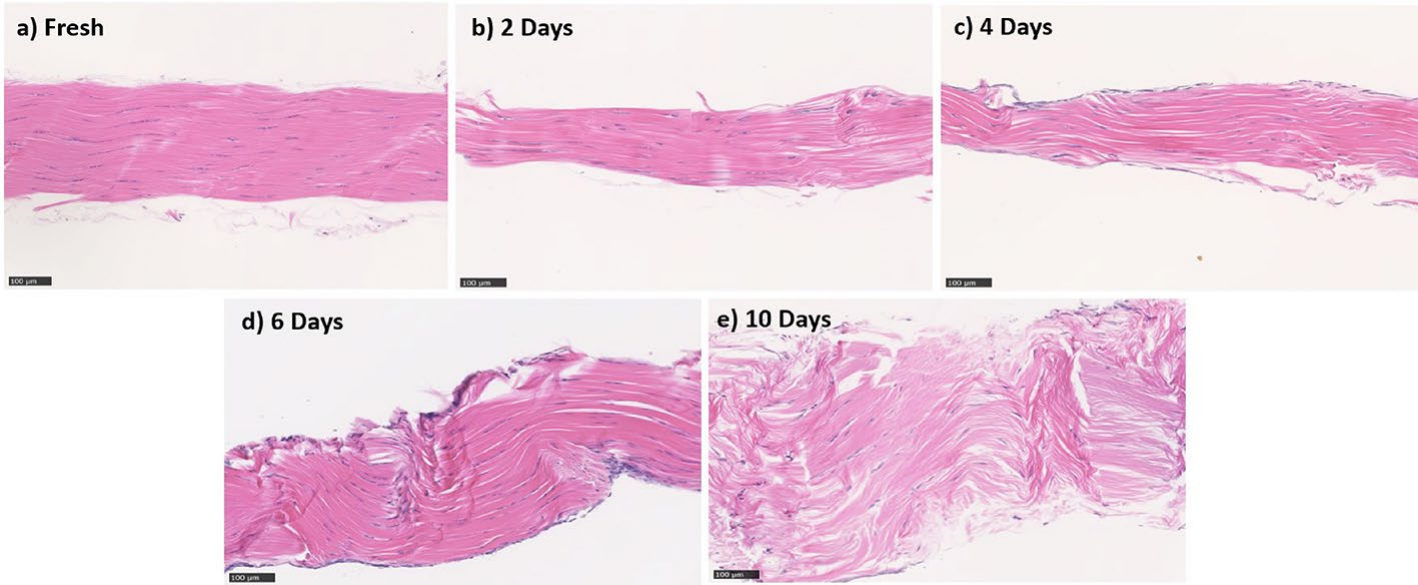
Typical micrographs of longitudinal tissue sections stained with hematoxylin and eosin showing pathological features of SD tendons. **a** freshly isolated, **b** 2 days SD, **c** 4 days SD, **d** 6 days SD, **e** 10 days SD. Pink = collagen fibers in connective tissues; Purple = tenocyte nuclei. Scale bar = 100 μm

Under light microscopy, freshly harvested samples showed dense and well-aligned collagen fibers (Fig. 4a). Following SD, tendons exhibited more loosely packed and wavy collagen fibers and had increased space area between fibers compared to the fresh control samples (Fig. 4 b–e). Moreover, under SD conditions, tendon cells started to lose their elongated shape and became more rounded in shape (Fig. 4b–e).

**Fig. 5:**
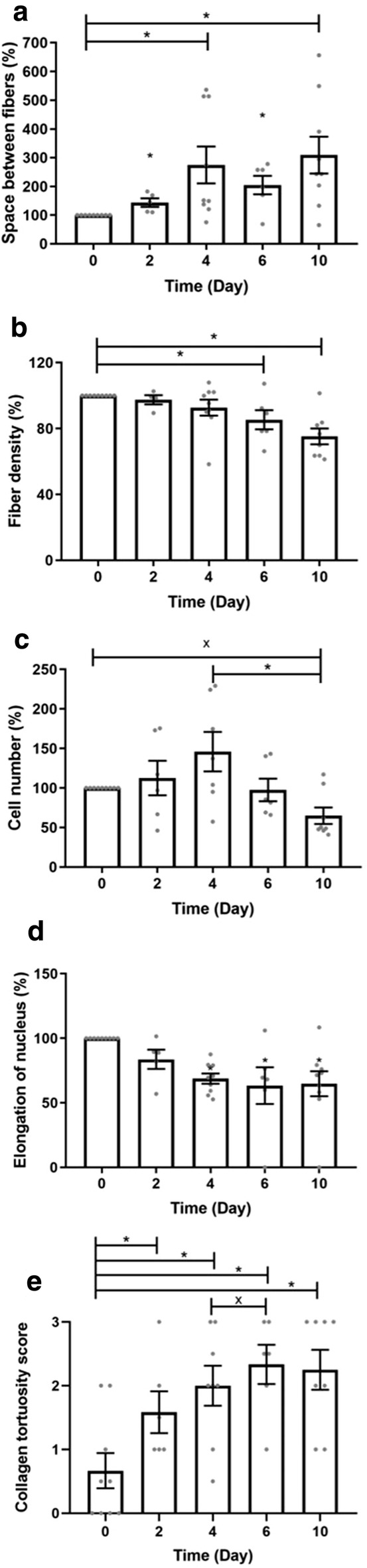
Quantification of **a** space between fibers, **b** fiber density, **c** cell number, **d** nucleus elongation, and **e** semiquantification of collagen tortuosity. The data are presented as the means and SEM. *p < 0.050 versus control fresh tendons; and ^×^*p* < 0.050 versus 4 days of stress deprivation. One-sample Wilcoxon tests were used to compare the groups to day 0. Nonparametric Wilcoxon matched-pairs signed rank tests were used to compare groups (except day 0) to each other

Quantitative data are presented in Fig. 5. Before normalization, the space area between fibers was evaluated at 42.5 ± 3.5 pixels (Fig. 5a). There was a significant increase in the space area at each time point compared to fresh tendons (until ~ 300% at day 10). For these samples, the fiber density was evaluated at 92.4 ± 0.9% before normalization (Fig. 5b). There was a significant decrease in fiber density after 6 and 10 days of SD compared to fresh tendons (to ~ 85% and ~ 75%, respectively).

Fiber tortuosity increased with culture time, as evaluated by the scoring technique. This increase was significantly different at days 2, 4, 6, and 10 of SD compared to day 0 (Fig. 5c). The cell number of fresh tendons was found to be 251.9 ± 19.7 cells/mm^2^ before normalization (Fig. 5d). Cell number first increased to 113% and 146% at days 2 and 4, respectively. It then decreased to 98% and 65% at day 6 and 10 of SD, respectively. The cell density at day 10 was significantly different from those on day 0 and day 4. The elongation of the nuclei of fresh tendons was evaluated to 2.3 ± 0.1 (Fig. 5e).

Nucleus elongation after 4, 6, and 10 days of SD decreased significantly compared to that of fresh tendons (to ~ 69%, ~ 63% and ~ 65% respectively).

### Immunohistochemistry results

**Fig. 6:**
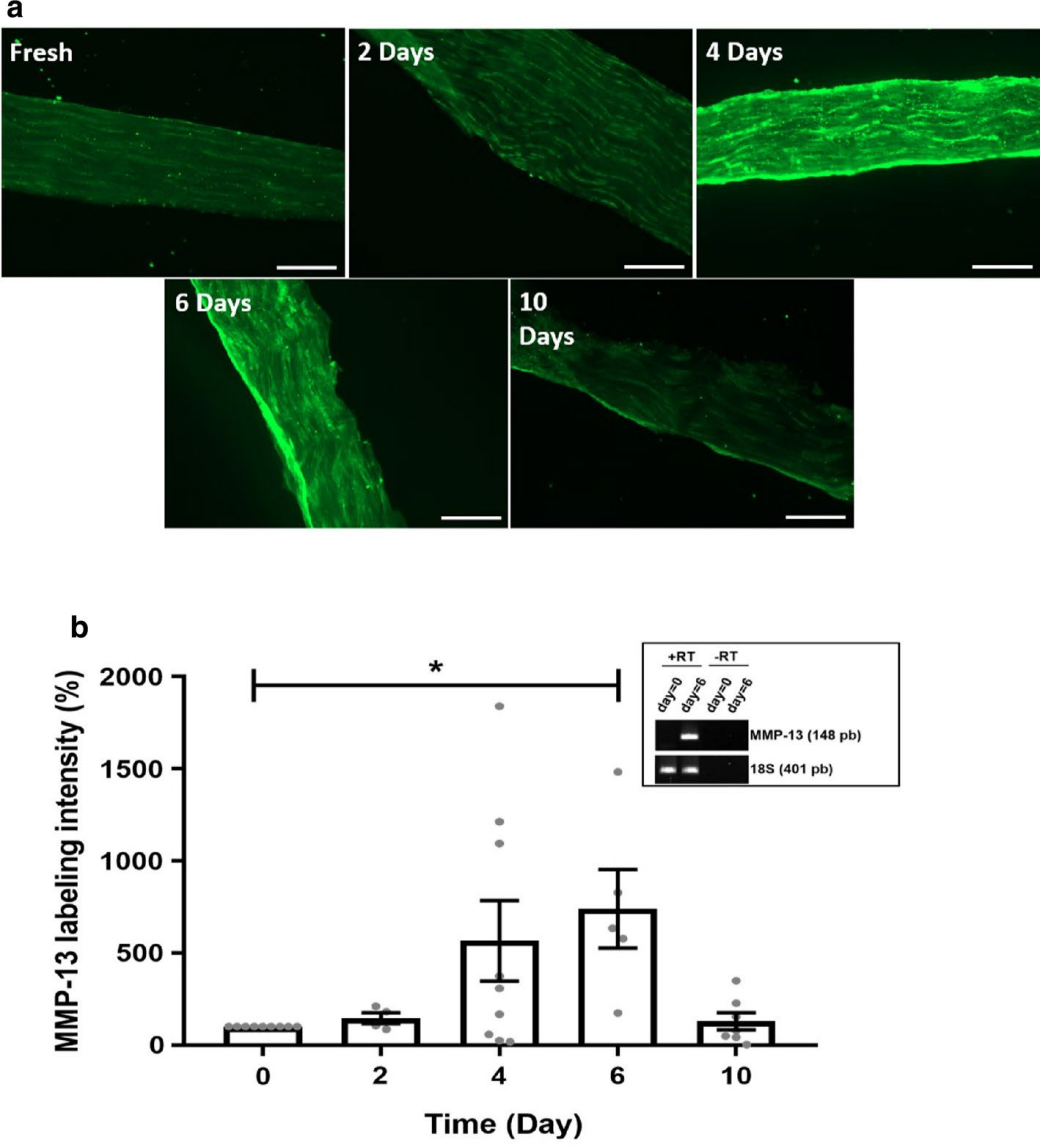
Immunohistochemistry analysis of MMP-13. **a** Representative epifluorescence micrographs of freshly isolated and SD tendons stained for MMP-13 protein using the rabbit polyclonal anti-MMP-13 antibody ab39012. Scale bar = 200 μm. **b** Bar graph illustrating semiquantitative MMP-13 immunofluorescent intensity in freshly isolated tendons and SD tendons. Inset: consolidation of increased MMP-13 in one tendon sample by PCR analysis. *p < 0.050 versus control fresh tendons

In fresh tendons, MMP-13 had an intensity level of 537.36 ± 358.5 (arbitrary unit). SD tendons demonstrated an upregulation of MMP-13 with increasing SD time compared to fresh tendons (Fig. 6a, b). Epifluorescent staining data revealed that this upregulation was statistically significant at day 6 compared to the fresh tendons (Fig. 6b). However, at day 10, there was a decrease in MMP-13 level to ~ 130% compared to that at the previous SD time point (day 6). This could possibly be explained by the increased cell death after this incubation time (see Fig. 2).

## Discussion

We further developed the currently utilized ex vivo tendinopathy model in RTTs to include moderate levels of tendinopathy. We conducted two sets of experiments: core experiments and secondary experiments. In core experiments, we demonstrated that, as hypothesized, moderate tendinopathy: (1) occurs at 4 days SD; and (2) is characterized by a progressive increase in cell apoptosis, cell roundness, and MMP-13 level, as well as a progressive decrease in fiber density. However, contrary to our hypothesis, cell density increased until day 4, and no significant change was observed in mechanical properties after 4 days of SD. Decrease in mechanical properties rather occur as of day 6. The secondary data allowed for stronger statistical analysis between days 0, 4, and 10. This model could be useful for studying the progression of physiopathology in mechanobiological studies as well as for testing potential treatments to stop or reverse the progression of the pathology. That is, for example to explore the effect of new drugs or combination of drugs in pharmacological studies or loading regimens in physical treatments such as mechanical loading, this model can be used. In this regard, there have been several reports describing the effects of drugs such as PRP [24–32] and MMPI [33–37] on damaged tendons. However, these studies were either conducted on surgically induced tendinopathies, or when conducted on chronic tendinopathy there was a lack of information about the stage of tendon degeneration. Therefore, it is necessary to evaluate the effect of potential treatments on moderate damaged tendons.

In both ex vivo and in vivo experiments, an increase in cell number has been reported in tendinopathic tendons [8, 38–41]. This hypercellularity appears to be the initial response of tendons to activate their repair mechanism [8]. However, cellularity decreases with further progression of tendinopathy [8, 39]. This results in an inverted-U curve relating cellularity with time. In our experiment, we found that peak cellularity occurs at 4 days of SD, which we refer to as moderate tendinopathy. In contrast to these observations, in vivo SD of rabbit patellar tendons resulted in a progressive increase in cells over a 6-week period [41]. One reason explaining these observations could be differences in the underlying mechanisms of tendinopathy between ex vivo and in vivo models.

Previous ex vivo studies (Table 1) have reported that short-term SD does not affect the biomechanical properties, which is in accordance with our results (Fig. 3). These studies highlight a lack of information regarding the time point at which significant degradation in biomechanical properties occurs. Our observations show that biomechanical properties are affected by SD only after approximately 5 days of culture time. The observed 5-day delay in mechanical response to SD could be related to the absence of mechanical degradation, i.e., degradation through mechanical loading, in disuse models of tendinopathy development.

One of the pathologic characteristics that tendons experience during tendinopathy development is cell apoptosis [8, 42, 43]. In agreement with the literature, we observed a decrease in live cells and an increase in dead cells in both short-term and long-term tendinopathies. However, we did not specifically look at the type of cell death. In addition to the results from short- and long-term SD, the current study also provides data from mid-term SD. In the current study, the results from day 4 of SD confirm that there is a continuous increase in cell death throughout the early, moderate, and advanced tendinopathy stages.

There is also evidence that tendon cells transform from an elongated shape to a round shape in tendinopathic tendons [2], in agreement with our observations. Based on previous ex vivo studies, cell roundness appears only after 24 h of SD [5] and increases with culture time. Our data suggest that this increase in early and advanced tendinopathies is continuous through moderate tendinopathy as well.

Our data suggest that in addition to cellular shape, density and viability, gene response is also affected by SD. In agreement with our results, increases in MMP-13 (mRNA and protein) have been consistently reported in tendinopathy [2, 5–7, 10]. In the current study, despite the initial increase, we observed a decrease in MMP-13 levels after 10 days of SD. This decrease may be due to a decline in cell density and/or cell viability after 10 days of SD.

We observed a progressive decrease in fiber density of SD tendons from early (short-term) to advanced (long-term) tendinopathy. This decrease in fiber density of tendons has also been reported in previous ex vivo studies examining short- and long-term SD [6, 8, 10]. An increase in the space area between fibers was also reported at both time points [6, 8].

Usually, changes in fiber density and the space area between fibers are accompanied by disorganized collagen bundles [2]. Our data suggest a continuous decrease in collagen alignment through early to moderate and advanced tendinopathy.

Currently, there is no single model available that represents all aspects of human tendinopathy [4, 44]. Similar to other models, SD models have limitations. During SD, tendon properties are altered without exposing the tendon to mechanical loading. For this reason, SD models do not include simultaneous enzymatic and mechanical degradation. Accordingly, the model used in this study should be used with caution when investigating overuse injuries.

On the other hand, it has been theorized that in both cases of underuse and overuse tendinopathy, cell undergoes understimulation [5]. Based on this theory, no matter what kind of loading is creating tendinopathy, the destructive mechanism probably is exposing cells to a local loss of homeostatic strain as a result of isolated, microscopic collagen fiber damage.

Still, SD models relate more closely to immobilization scenarios such as casting or rest after surgery. Indeed, applying mechanical loads to SD tendons has been used to deepen our understanding of rehabilitation in these situations [8]. Finally, since SD models are easy to implement, they could be an excellent choice for studies in which multiple samples and conditions are needed. It should be noted that our SD system and obtained results were duplicable for 4- to 6-month-old rats. However, Lavagnino et al. [45] observed age-related changes in the behavior of 7-day SD tendons. It is also probable that changing the species, strain, or sex of the animals would alter some of the results, such as the timescale of the tendinopathy development. Moreover, we investigated tendon properties with 2-day intervals. Studying 5-day SD tendons would also allow for a more detailed characterization of moderate tendinopathy. These could be the subject of future investigations.

Also, obviously, these ex vivo results will have to be consolidated with in vivo data obtained from recognized models of tendinopathy.

## Conclusions

In conclusion, we have further characterized a currently utilized ex vivo model to develop moderate tendinopathy in RTTs using the SD method. To our knowledge, this is the first study to characterize the moderate stage of tendinopathy. Our data suggest that moderate tendinopathy occurs around day 4 of SD. Because advanced tendinopathy can be more difficult to cure and because early tendinopathy is often asymptomatic, meaning that patients most likely do not seek medical treatment, it is reasonable to test treatments for moderate tendinopathy first and, if successful, to proceed with treatments for more advanced tendinopathy. Our study identified a moderate stage of tendinopathy in an ex vivo SD model based on the viability, pathohistology, immunohistochemistry, and biomechanical properties of tendons.
